# Associations between type of childhood adversities and labour market participation and employment conditions in young adults

**DOI:** 10.1136/jech-2022-219574

**Published:** 2023-02-17

**Authors:** Tjeerd Rudmer de Vries, Iris Arends, Albertine J. Oldehinkel, Ute Bültmann

**Affiliations:** 1 Department of Health Sciences, Community and Occupational Medicine, University Medical Center Groningen, University of Groningen, Groningen, The Netherlands; 2 Arbo Unie BV, Utrecht, The Netherlands; 3 Department of Psychiatry, Interdisciplinary Center Psychopathology and Emotion Regulation, University Medical Center Groningen, University of Groningen, Groningen, The Netherlands

**Keywords:** public health, employment, life course epidemiology, education, unemployment

## Abstract

**Background:**

Cumulative exposure to childhood adversity is associated with a variety of labour market outcomes in young adulthood. It remains largely unclear whether the type of adversity matters in this association. This prospective study examined the differences in exposure to 14 adverse experiences among groups of young adults aged 22 characterised by distinct labour market participation states and employment conditions.

**Methods:**

We used data from the TRacking Adolescents’ Individual Lives Survey, a Dutch prospective cohort study with 15 years of follow-up (N=1524). We included 14 adverse experiences (ages 0–16) across five domains: peer influences, loss or threat of loss, material deprivation, family dynamics and maltreatment. Labour market participation states and employment conditions were assessed at age 22. We used latent class analysis to derive labour market outcome groups, which we subsequently compared on exposure to adverse experiences using pairwise comparisons.

**Results:**

Inactive individuals (n=85, 5.6%), often neither in education (77.4%) nor employment (98.6%) and on benefits (94.4%), were more likely to be exposed to many distinct types of adverse experiences (eg, parental addiction, bullying victimisation) as compared with all other groups. Early workers (n=413, 27.1%), often on temporary contracts and low monthly incomes, were more likely to be exposed to parental divorce (22.7%) compared with students with side jobs (12.9%).

**Conclusions:**

Different adverse experiences are not equally associated with labour market outcomes. Researchers and stakeholders in policy and practice should be aware of the differences between adverse experiences in their importance for labour market outcomes in young adults.

**What is already known on this topic**

- Current evidence suggests that cumulative exposure to childhood adversity is associated with early labour market entry and labour market inactivity in young adulthood.
- It remains unclear whether the type of childhood adversity matters (eg, experiences of maltreatment vs experiences of material deprivation) and whether childhood adversity exposure matters for labour market outcomes beyond labour market participation, namely employment conditions such as contract types, work hours and monthly income.

**What this study adds**

- Our study reveals important nuances in the association between childhood adversity and labour market outcomes in early working life.
- We found that different groups of young adults, characterised by distinct labour market participation states and employment conditions, vary markedly in their exposure to types of adverse experiences.

**How this study might affect research, practice or policy**

- Future research on childhood adversity and labour market outcomes should continue disentangling the different types of adversity to facilitate translating findings to policy and practice.
- Stakeholders in policy and practice may benefit from an increased awareness of the differences between adverse experiences in their importance for labour market outcomes in young adults to improve labour market outcomes at the population level.

## Introduction

Childhood adversity, which includes adverse experiences (AEs) such as financial difficulties, parental mental illness and bullying victimisation, is common around the world.^1–3^ AEs are defined as experiences that require significant adaptation by the developing child in terms of psychological, social and neurodevelopmental systems, and are outside the normal expected environment.^4^ Childhood adversity is an important determinant of health inequalities throughout the life course.^5 6^ Employment in young adulthood has been suggested as an important driver of these health inequalities,^7–9^ but only a few studies have examined the association between childhood adversity and employment outcomes in young adulthood thus far.

Studies to date have shown that cumulative exposure to childhood adversity is associated with a variety of outcomes relevant to young adults’ working lives: unemployment,^10 11^ benefit recipiency,^12^ income,^13^ NEET (not in employment, education or training) and labour market participation trajectories.^14–17^ However, cumulative exposure conceptualisations of childhood adversity have been criticised for not providing evidence that is required to better understand the mechanisms through which adversity is associated with outcomes of interest. Developing mechanistic insight requires knowledge on the role of individual AEs.^18^ Most recently, Hansen *et al* 15 showed that young adults whose working lives were characterised by early full-time employment throughout young adulthood after obtaining a vocational degree, or by reduced labour market participation (eg, frequent or long-term unemployment spells), largely differed in the type of exposure to adversity compared with young adults who were studying for most of their early 20s. Especially those AEs that relate to behaviours of parents, such as abuse and parental addiction, were strongly associated with frequent or long-term unemployment spells; AEs such as parental divorce and witnessing violence were strongly associated with early labour market entry.^15^ AEs that more or less befall parents, such as a physical illness or a death of a parent, were not associated with labour market participation in young adulthood.^12 15^ Only few other studies investigated the importance of individual AEs for labour market outcomes, and there is marked between-study heterogeneity with regard to assessments of AEs and labour market outcomes. As a result, current evidence on the importance of individual AEs for labour market outcomes is fragmented and remains limited.^12 14–16^

Currently available evidence suggests that it is possible to distinguish between groups of young adults on the labour market, which may be differentiated in terms of labour market participation states, employment conditions and adversity exposure patterns.^15^ These groups may require diverging policy strategies tailored to differences in exposure to adversity, as well as to the mechanisms through which adversities are associated with labour market participation and employment conditions. For example, young adults who experience maltreatment might be more often unemployed and on benefits due to ongoing mental health problems,^16^ whereas those who experienced material deprivation might enter the labour market earlier due to lower educational attainment.^19^ Providing policy makers with the necessary information to develop tailored strategies requires empirical evidence of labour market outcomes groups among young adults and how these groups differ in exposure to AEs.

In this study, we aimed to examine the associations between individual AEs and labour market outcomes in young adults aged 22. We extend the literature in two ways. First, we derived labour market outcome groups in young adults aged 22 through latent class analysis (LCA). These labour market outcome groups were based on labour market participation states, that is, employment, educational status, benefit status, and employment conditions, that is, monthly income, contract type and work hours. Second, we included a broad array of AEs in both the family and peer context to provide more conclusive evidence on the importance of individual AEs for labour market outcomes. Given the explorative nature of this study, we have not specified a priori hypotheses. The findings of this study may help identify groups of young adults who require tailored support in their early working lives, conditional on the type of childhood adversity exposure in childhood.

## Methods

### Sample and design

We analysed data from 1524 individuals included in the TRacking Adolescents’ Individual Lives Survey (TRAILS) cohort (68.4% of the baseline cohort), a Dutch prospective cohort study with biennial measurement waves. In March 2001, a representative sample of 2935 children born in one of the three northern provinces of the Netherlands between 1 October 1989 and 30 September 1991 were approached, of whom 2229 children (76%) enrolled in the study at baseline. We used data from the first, second, fourth and fifth waves of TRAILS. The mean ages of the respondents were 11.08 years (SD=0.56), 13.53 years (SD=0.52), 19.02 years (SD=0.58) and 22.26 years (SD=0.65) across waves, respectively. Individuals who were lost to follow-up (n=705, 31.6%) were more likely to be male (Z=−7.28, p<0.001) and to have parents with low educational level (Z=−10.41, p<0.001). More indepth information about TRAILS has been presented elsewhere.^20–22^ Informed consent was obtained from all participants and their parents.

### Measures

#### AEs between ages 0 and 16

We included 14 AEs across five domains, akin to Rod *et al* 23: peer influences (bullying victimisation, peer rejection), loss or threat of loss (familial death, illness of a sibling, physical illness of a parent), family dynamics (parental divorce, familial conflicts, parental mental health problems, parental addiction), material deprivation (parental unemployment, financial difficulties) and maltreatment (sexual abuse, physical abuse, emotional abuse). Answer categories for all AEs were 0: not exposed and 1: exposed. Exposure to most of the AEs was reported by parents (apart from bullying victimisation, peer rejection, and abuse). A detailed description of the AEs with information on measurement instruments, the timing of measurement, the reporter and the observation period is provided in online supplemental data 1 and table S1.

10.1136/jech-2022-219574.supp1 Supplementary data

#### Labour market participation states and employment conditions at age 22

Three labour market participation states were considered: educational status (not currently studying, following a high school education, currently pursuing a vocational degree, currently pursuing a (applied) university degree), employment status (unemployed, employed, self-employed) and benefit status (receiving benefits yes/no). Three employment conditions were considered: contract type (permanent contract, temporary contract, temporary employment agency contract), work hours (12–35 hours, >35 hours) and monthly income (€300 or less, between €301 and €600, between €601 and €900, between €901 and €1200, and €1201 or higher). A detailed description of the labour market participation states and employment conditions, including rationales for specific categorisations, is provided in online supplemental data 2.

#### Control variables

Parental educational level at baseline was included to adjust for confounding.^12 15 24 25^ Parental educational level was classified into low (primary, lower vocational and lower secondary education), medium (intermediate vocational, intermediate secondary education and higher secondary education) and high (higher vocational education and university).^26^ The highest educational level across both parents was considered.

#### Demographic characteristics

We included sex (male/female) at baseline to further describe our study sample.

### Statistical analysis

Labour market outcome groups were derived using LCA,^27^ using the following variables: educational status, a combined employment status and contract type variable (unemployed, employed with a permanent contract, employed with a temporary contract, employed with a temporary employment agency contract, self-employed), benefit status, work hours and monthly income. For the purpose of the analysis, the work hours variable was complemented with two additional categories (side jobs, reflecting 1–12 hours work and 0 hours to align with the unemployment category of the employment status variable). Five different class solutions were estimated (ranging from two to six classes). Model fit (global) was assessed through the Bayesian information criterion and Akaike information criterion metrics. Model interpretability and entropy (class separability) were also considered. The optimal class solution was (parametrically) bootstrapped to assess local model fit through an investigation of bivariate residuals between variables included in the LCA. Direct effects were included to address any violations of the local independence assumption.^28^ The four-class solution was found to be most optimal. This decision was mainly based on class separation and interpretability, as the fit statistics of the four-class solution were similar to the five-class and six-class solutions (see online supplemental table S2).

To investigate whether exposure to the AEs significantly differed across the labour market outcome groups (overall effect), we included each AE to the latent class solutions separately. In case of a significant overall effect (alpha of 0.05), pairwise comparisons (Wald tests) were conducted to ascertain group-specific differences (alpha of 0.05). We present conditional probabilities of exposure to each AE given class membership from these multinomial logistic regression models as well as gamma coefficients (due to the use of effects coding as opposed to reference category coding) and their SEs. Pairwise comparisons were adjusted for multiple testing with the Benjamini-Hochberg procedure (false discovery rate (FDR) procedure).^29^ We set the fixed FDR to 5%, indicating that 5% of all significant findings were allowed to be false-positive. All analyses included parental education to adjust for confounding. We used proportional class assignment with maximum-likelihood-based (ML) adjustments to avoid downwards-biased estimates (ie, the bias-adjusted three-step approach).^30^ Missing data were considered missing at random and handled using full information maximum likelihood in all analyses (see online supplemental table S5 for an overview of missing data across all study variables). All analyses were conducted with the Latent GOLD 6.0 software.^31^

### Sensitivity analyses

We re-examined the associations between AEs and labour market outcome groups using the six-class solution and found relatively few differences as compared with the four-class solution (see online supplemental tables S3 and S4). Comparisons with the three-class and five-class solutions were not performed due to multiple violations of the local independence assumptions in these solutions.

## Results

Most respondents were female (56.3%) and had parents with a medium educational level (62.9%). The prevalence rates of AEs ranged from 2.6% (parental addiction) to 25.9% (parental physical illness) (see table 1).

**Table 1:** Sample characteristics

Variable	Total (N=1524)
Female, n (%)	858 (56.3)
Parental educational level, n (%)	
Low	283 (18.9)
Medium	944 (62.9)
High	275 (18.3)
Adverse experiences, n (%)	
Bullying victimisation	106 (7.1)
Peer rejection	71 (4.8)
Familial death	41 (2.7)
Parental divorce	276 (18.5)
Familial conflicts	97 (6.9)
Parental unemployment	115 (9.6)
Financial difficulties	67 (4.7)
Sibling illness	137 (9.2)
Parental illness	385 (25.9)
Parental mental health problems	310 (21.5)
Parental addiction	38 (2.6)
Sexual abuse	64 (4.5)
Physical abuse	62 (4.4)
Emotional abuse	198 (13.8)

### Labour market outcome groups at age 22

Table 2 contains the conditional probabilities of the labour market outcome variables across each of the four labour market outcome groups (latent classes). The four groups were labelled ‘students with side jobs’ (n=640, 41.2%), ‘early workers’ (n=413, 27.1%), ‘non-working students’ (n=386, 25.3%) and ‘inactive individuals’ (n=85, 5.6%), respectively.

**Table 2:** Labour market outcome variables at age 22, total and conditional on labour market outcome groups

Variable	Total N=1524, n (%)	Students with side jobs n=640 (%)*	Early workers n=413 (%)*	Non-working students n=386 (%)*	Inactive individuals n=85 (%)*
Educational status					
Not in education	521 (34.2)	4.1	90.4	14.2	77.4
Lower education	8 (0.5)	0.3	0.8	0.6	1.0
Vocational education	175 (11.5)	12.7	4.8	17.1	9.4
Applied science or university	820 (53.8)	82.9	4.0	68.1	12.3
Employment status					
Unemployed	467 (30.7)	0.0	0.0	99.5	98.6
Employed with permanent contract	496 (32.7)	49.2	44.4	0.0	0.1
Employed with temporary contract	351 (23.1)	33.4	33.6	0.0	0.1
Employed with temporary employment agency contract	163 (10.7)	14.1	17.3	0.5	0.0
Self-employed	41 (2.7)	3.2	4.7	0.0	1.2
Benefit status	98 (6.43)	0.2	5.4	0.0	94.4
Work hours/week					
0 hours	470 (30.9)	0.1	0.0	99.9	99.6
Between 1 and 12 hours (side job)	425 (28.0)	61.2	8.2	0.4	0.4
Between 12 and 35 hours (part time)	421 (27.6)	35.4	47.5	0.0	0.0
>35 hours (full time)	204 (13.4)	3.4	44.4	0.0	0.0
Monthly income (€)					
<300	241 (16.5)	19.4	2.1	30.3	5.3
301–600	409 (28.0)	34.1	9.8	39.3	19.6
601–900	361 (24.7)	27.3	21.7	21.9	30.4
901–1200	258 (17.6)	13.8	31.3	7.0	27.2
>1201	194 (13.3)	5.4	35.2	1.5	17.6

*Conditional on group membership.

### Type of AEs between ages 0 and 16 and labour market outcome groups at age 22

The labour market outcome groups differed significantly in exposure to nine AEs across domains: bullying victimisation, peer rejection, parental divorce, familial conflicts, financial difficulties, parental addiction, sexual abuse, physical abuse and emotional abuse. A total of 29 pairwise comparisons (out of 54) were significant (table 3). The majority of the significant pairwise comparisons pertain to differences between the inactive individuals group and the other groups, with the inactive individuals group being significantly more likely to be exposed to each of the nine AEs compared with the other groups (with the exception of financial difficulties, for which the inactive group only differed from the students with side jobs). The early workers group had significantly higher exposure rates to parental divorce than students with side jobs (22.7% vs 12.9%).

**Table 3:** Distribution of adverse experiences between ages 0 and 16, conditional on labour market outcome groups at age 22

Adverse experiences	Students with side jobs (%)	Early workers (%)	Non-working students (%)	Inactive individuals (%)	SIGNIFICANT PAIRWISE COMPARISONS‡
Peer influences					
Bullying victimisation	4.8	7.4	6.2	24.8	1–4, 2–4, 3–4
Peer rejection	3.2	4.6	5.0	14.9	1–4, 2–4, 3–4
Loss or threat of loss					
Familial death	2.6	2.7	1.6	8.0	*
Illness of a sibling	7.5	8.7	11.6	10.3	*
Illness of a parent	25.3	26.3	23.2	29.8	*
Material deprivation					
Parental unemployment	6.1	6.0	7.4	6.9	*
Financial difficulties	3.2	5.1	4.1	11.4	1–4, 2–4†
Family dynamics					
Familial conflicts	5.3	7.3	5.8	21.2	1–4, 2–4, 3–4
Parental divorce	12.9	22.7	15.8	45.8	1–4, 2–4, 3–4, 1–2
Parental mental health	19.1	20.1	19.7	33.7	*
Parental addiction	1.5	2.9	1.9	11.0	1–4, 2–4, 3–4
Maltreatment					
Sexual abuse	2.7	4.4	4.5	2.7	1–4, 2–4, 3–4
Physical abuse	3.1	3.6	3.9	14.2	1–4, 2–4, 3–4
Emotional abuse	10.7	14.4	12.9	24.2	1–4, 2–4, 3–4

*Not included in the pairwise comparison analyses due to non-significant omnibus test.

†Likely to reflect a false-positive finding.

‡ Numbers depicted in this column refer to the four labour market group, from left to right, respectively.

The four groups did not differ in exposure to any of the AEs belonging to the domain of loss or threat of loss, parental unemployment and parental mental health problems. Table 4 provides the estimates for the magnitude of the associations between AEs and the four labour market outcome groups.

**Table 4:** Parameter estimates (gamma coefficients and their SEs) for the associations between adverse experiences between ages 0 and 16 across the four labour market outcome groups at age 22

Adverse experiences	Students with side jobs	Early workers	Non-working students	Inactive individuals
Peer influences				
Bullying victimisation	−0.32 (0.08)	−0.11 (0.09)	−0.18 (0.11)	0.60 (0.17)
Peer rejection	−0.31 (0.10)	−0.13 (0.10)	−0.08 (0.11)	0.52 (0.13)
Loss or threat of loss				
Familial death	−0.07 (0.13)	−0.08 (0.15)	−0.32 (0.19)	0.47 (0.17)
Illness of a sibling	−0.10 (0.08)	−0.06 (0.09)	0.16 (0.08)	−0.01 (0.13)
Illness of a parent	−0.01 (0.05)	0.02 (0.06)	−0.07 (0.06)	0.09 (0.09)
Material deprivation				
Parental unemployment	−0.12 (0.07)	−0.10 (0.08)	−0.02 (0.08)	0.25 (0.12)
Financial difficulties	−0.22 (0.10)	−0.07 (0.10)	−0.05 (0.11)	0.33 (0.13)
Family dynamics				
Familial conflicts	−0.32 (0.08)	−0.07 (0.09)	−0.17 (0.09)	0.55 (0.12)
Parental divorce	−0.28 (0.06)	−0.04 (0.06)	−0.13 (0.07)	0.44 (0.09)
Parental mental health	−0.08 (0.06)	−0.10 (0.06)	−0.05 (0.06)	0.23 (0.09)
Parental addiction	−0.33 (0.12)	−0.08 (0.12)	−0.19 (0.14)	0.58 (0.15)
Maltreatment				
Sexual abuse	−0.32 (0.10)	−0.11 (0.10)	−0.04 (0.11)	0.47 (0.13)
Physical abuse	−0.24 (0.10)	−0.21 (0.10)	−0.11 (0.11)	0.56 (0.13)
Emotional abuse	−0.19 (0.07)	−0.03 (0.07)	−0.07 (0.07)	0.29 (0.10)

Note: Row estimates sum up to approximately 0 (slight deviations are due to rounding). The presented coefficients are for exposure (1=exposed). To obtain coefficients for the reverse (0=non-exposed), the respective sign for each estimate is to be reversed. Estimates within categories (1=exposed and 0=non-exposed) sum up to 0. Positive coefficients indicate that a combination of variables (here: exposure to a respective adverse experience and group membership) is more likely to occur than average; negative coefficients refer to the opposite.

## Discussion

Our results show four distinct labour market outcome groups in young adults aged 22, students with side jobs, early workers, non-working students and inactive individuals, which differ in type of childhood adversity exposure. Exposure across several domains of childhood adversity was significantly more likely among the inactive individuals group as compared with all other groups. We also found that exposure to parental divorce was significantly more likely among the early workers group compared with students with side jobs. We found no evidence that the labour market outcome groups differed in exposure to any of the AEs belonging to the domain of loss or threat of loss, parental unemployment and parental mental health problems. Our results suggest that inactive individuals differ from the other groups in exposure to a wide variety of AEs, both in the family (eg, parental addiction) and peer contexts (eg, bullying victimisation). Differences in exposure to AEs among the other groups are less pronounced and limited to parental divorce.

Across studies, it becomes apparent that exposure to especially AEs that pertain to direct psychological (eg, bullying victimisation, peer rejection, emotional abuse) or physical harm (physical abuse), or increase the likelihood thereof (eg, parental addiction, conflicts in the family, financial difficulties), is very high among individuals whom we characterised as inactive individuals in this study.^12 14–16^ One likely explanation for this finding is that exposure to the aforementioned AEs is strongly associated with increased mental health problems,^32 33^ which in turn have been demonstrated to be associated with unemployment in young adulthood.^34^

Our findings suggest that students with side jobs, early workers and non-working students largely do not differ in exposure to childhood adversity exposure, despite differences in labour market participation states and employment conditions. Similar to Hansen *et al*,^15^ we found that exposure to parental divorce was more likely among young adults with an early labour market entry than among students with side jobs. Parental divorce has been shown to be associated with school dropout and lower educational attainment, which might explain why the exposure to parental divorce was higher among the early workers than among students.^35^ Unlike Hansen *et al*,^15^ we did not find that the early workers were also more exposed to abuse and parental addiction. It is possible that this difference is attributable to differences in assessment since we used parental report, whereas Hansen *et al* 15 used adult self-report; adult self-report of childhood adversity has been shown to be more strongly associated with outcomes than parental report.^36 37^ The impact of differences in reporters (eg, caregiver, self-report, register-based assessment) warrants attention in future research.

This study has several strengths. TRAILS’ longitudinal design made it possible to investigate the association between types of AEs and labour market outcomes in young adults in a prospective fashion. The richness of the TRAILS cohort also allowed us to include a broad array of AEs with relatively short recall periods in the family and peer contexts, including often-ignored AEs such as financial difficulties and bullying victimisation.^38 39^ This study is also the first to confirm and extend previous findings from primarily Scandinavian studies with data from the Netherlands.^12 14 15^ Nevertheless, the findings should be interpreted considering several limitations. First, although TRAILS has only limited loss to follow-up, the study is subject to selection bias due to attrition. Missing data analyses showed that men and participants with parents with lower educational level were more likely to not participate over time. Because exposure to childhood adversity was more likely to occur in families of lower socioeconomic background (results not shown), the findings of this study may underestimate the differences in exposure to childhood adversity among the four labour market outcome groups (see also Walsh *et al* 25). Studies with data on more individuals from a lower socioeconomic background may provide more insight into the generalisability of our findings. Second, due to the relatively small sample size of this study, it is possible that smaller differences in exposure to AEs between the four labour market outcome groups were not uncovered due to low statistical power (although these differences might be negligible and thus irrelevant). We are confident however that most differences between the inactive individuals group and the other groups were uncovered because these groups differed drastically in their exposure to the included AEs.

Our findings have several implications for research, policy and practice. Our study highlights that especially maltreatment and peer influences, and family dynamics and material deprivation to some extent, are important for labour market outcomes in young adulthood. We recommend practitioners and policy makers to focus specifically on those AEs that may be regarded as psychologically or physically harmful (eg, abuse, peer victimisation), or AEs that increase the likelihood thereof (eg, parental addiction, conflicts in the family, financial difficulties), as those AEs are particularly important for labour market inactivity early in young adults’ working careers. Nevertheless, it is also important to not overlook individuals who are exposed to a few specific AEs (eg, parental divorce) and who are likely to be employed in young adulthood. These individuals require different support from individuals who are inactive on the labour market. We have three important recommendations for future research. First, future studies should explore the mechanisms that explain the associations between specific AEs and labour market outcomes. It is likely that the different labour market outcome groups are affected by childhood adversity through different mechanisms. Elevated and ongoing mental health problems and school dropout are likely to play a prominent role for especially inactive individuals.^16 32 34 35^ Divorced parents of early workers might have fewer resources to support their children in their educational careers, which may lead to young adults to discontinue studying after obtaining a low-level qualification and start working thereafter.^19^ Understanding through which mechanics AEs, both individually and conjunctively, affect labour market outcomes may be vital to reduce the long-term impact of childhood adversity. Second, future studies may investigate the role of co-occurrence of AEs, preferably including information on the timing, frequency or severity,^18^ that were predictive of labour market outcomes. Doing so may provide more fine-grained insight into exposure patterns of the various labour market outcome groups described in this study. Third and last, future studies should further investigate to what extent labour market outcomes in young adulthood mediate the association between childhood adversity and health inequalities throughout the life course, taking into account the differences in exposure patterns among different groups of young adults.^7–9^ To conclude, our findings show that the type of AEs matters in labour market outcomes in young adults. Stakeholders in policy and practice may benefit from an increased awareness of the differences between AEs in their importance for labour market outcomes in young adults when aiming to reduce the occurrence of childhood adversity and its potential consequences for labour market outcomes at the population level.

## Data Availability

Data may be obtained from a third party and are not publicly available. TRAILS data of the T1, T2, T3, T4 and T5 measurement waves are deposited in the Data Archiving and Networked Services of the Royal Dutch Academy of Sciences (DANS-KNAW) and access can be requested at http://www.dans.knaw.nl.
