# Supplementary material for: Associations between type of childhood adversities and labour market participation and employment conditions in young adults
Source: J Epidemiol Community Health. 2023 Feb 17;77(4):230–6. doi: 10.1136/jech-2022-219574 (PMC10086506; doi:10.1136/jech-2022-219574)
Supplement: Supplementary data [file jech-2022-219574supp001.pdf]

Supplementary material

This is the supplementary material belonging to the article entitled "Associations between Type of Childhood Adversities and Labour Market Participation and Employment Conditions in Young Adults". The supplement provides information regarding the following topics: 1) a description of the AEs with information on measurement instruments, the timing of measurement, the reporter, and the observation period is provided. This also includes a detailed description of the adverse experiences that were included in this study, 2) a detailed description of the labour market participation states and employment conditions that were included in this study, 3) information on fit statistics pertaining to the latent class analysis performed to derive labour market outcome groups in this study as well as information pertaining to the sensitivity analysis, and 4) an overview of missing data for the key variables used in this study (childhood adversities, labour market participation states and employment conditions).

Supplement 1. Measurement of adverse experiences

Table S1. Measurement of childhood adverse experiences

Adversity	Assessment waves	Age range(s)
Bullying	1 & 2*	10-11, 12-13
Peer rejection	1 & 2*	10-11, 12-13
Familial death	1±	0-12
Parental illness	1±	0-12
Parental mental health problems	1±	10-11
Parental addiction	1±	10-11
Sibling illness	1±	0-12
Parental unemployment	1±	0-12
Parental divorce	1±	0-12
Financial difficulties	2±	0-14
Familial conflicts	2±	0-14
Sexual abuse	4*	0-16
Emotional abuse	4*	0-16
Physical abuse	4*	0-16

± = Parental informant

* = Respondent

Detailed description of adverse experiences

Bullying victimisation. Information on bullying victimisation was obtained with the item "I am often bullied" of the Youth Self-Report (YSR) questionnaire [1]. Respondents were asked to indicate whether this statement was not true, (0), somewhat or sometimes true (1), or very true or often true (2) in the past six months. A score of two was considered indicative for being bullied.

Peer rejection. Information on peer rejection was obtained with the item 'Other boys or girls do not like me' of the YSR questionnaire [1]. A score of two was considered indicative for being rejected by peers.

Parental mental health problems. Information on the occurrence of parental mental health issues was obtained through vignettes [2,3] describing depressive complaints, anxiety, problem behaviour, and psychotic complaints. Both parents indicated whether they had experienced any of the aforementioned mental health problems in the past year. Maternal and paternal mental health problems were collapsed into one parental mental health problem variable.

Parental addiction. Information on the occurrence of parental addiction was obtained through vignettes [2,3] describing alcohol, drug- and gambling addictions. Both parents indicated whether they had experienced any of the aforementioned addictions in the past year. Maternal and paternal addiction were collapsed into one parental addiction variable.

Parental physical illness. Information on the occurrence of parental illness (chronic- or acute physical illness) was obtained with the Developmental History Interview (DHI, composed by TRAILS). Parents were asked to indicate whether the child had experienced a severe physical illness of the mother or father. Maternal and paternal illness were collapsed into one parental illness variable.

Illness of a sibling. Information on the occurrence of an ill-struck sibling was also obtained through the DHI. Parents were asked to indicate whether a brother or sister of the participant had experienced a severe illness.

Conflicts within the household. Information on the occurrence of conflicts within the household was obtained with the Long-Term Difficulties (LTD) questionnaire (composed by TRAILS) [4]. Parents were asked whether people within the household (in which the child

was not directly involved) had long-lasting conflicts with each other. Parents also indicated how old the child was when these conflicts started.

Death within the household. Information on the occurrence of a death within the family was obtained through the DHI. Parents were asked whether the child had experienced the death of anyone in their direct family (within or outside of the home environment), and when said death occurred. The type of family members considered in this study were limited to biological or adoptive parents and siblings.

Parental separation. Information on the occurrence of a divorce or separation between parents was obtained with the DHI [5]. Parents were asked to indicate whether the child had experienced the separation or divorce of the parents, and how old the child was when this event occurred.

Financial difficulties. Information on the occurrence of financial difficulties was obtained with the LTD questionnaire [6]. Parents were asked whether the family had financial difficulties, and how old the child was when the financial difficulties started.

Parental unemployment. Information on the occurrence of parental unemployment was obtained with the DHI. Parents were asked to indicate whether either parent were unemployed and since when they were unemployed.

Emotional abuse. Information on the occurrence of sexual abuse was obtained with the Traumatic Youth Experiences questionnaire (TYE, composed by TRAILS) [7]. Respondents were asked to indicate whether either parent shouted, screamed or yelled at them, whether either parent called them stupid, lazy or anything similar or whether either parent cursed or swore at them before the age of 16 and how often this occurred. Answer categories included never, once or twice, sometimes, often and very often. Emotional abuse was said to have occurred when any one of the indicators of emotional abuse was said to occur often or very often.

Physical abuse. Information on the occurrence of physical abuse was obtained with the TYE questionnaire [7]. Respondents were asked to indicate whether either parent hit them with an object, whether either parent hit them (very) hard with their fist or kicked them, whether either parent hit them with bear hands, whether either parent shook or squeezed them, or whether either parent assaulted them (repeatedly beat them very hard) before the age of 16 and how often this occurred. Answer categories included never, once or twice, sometimes,

often and very often. For assault, all answer categories, except from ‘never’, were classified as physical abuse. For the other indicators, often and very often were classified as physical abuse.

Sexual abuse. Information on the occurrence of sexual abuse was obtained with the TYE questionnaire [7]. Respondents were asked to indicate whether a family member, family acquaintance or a stranger either showed their genitals or masturbated before them, touched them in a sexual manner, coerced or forced them to touch the perpetrator in a sexual manner or had intercourse with them before the age of 16. Answer categories included never, yes this happened once before the age of 16 and yes this happened multiple times before the age of 16. Any occurrence was classified as sexual abuse.

Supplement 2 - Detailed description of labour market participation states and employment conditions

Labour market participation states

Educational status. Educational status (not currently studying, following a high school education, currently pursuing a vocational degree, and currently pursuing an (applied) university degree) was obtained by asking individuals whether they were currently in education, and what type of education they pursued.

Employment status. Employment status (unemployed, employed, self-employed) was determined by asking participants whether they were employed in the previous month.

Benefit status. Benefit status (not receiving benefits and receiving unemployment benefits, social assistance benefits or disability benefits) was obtained by asking whether participants received any benefits and what type of benefit this concerned. Individuals may claim unemployment benefits when they have lost their job through no fault of their own. In addition, individuals must have worked at least 26 weeks in the past 36 weeks to be eligible for unemployment benefits. Individuals may claim social assistance benefits when they do not have enough income to get by (i.e. their —joint— income must be below the social assistance standard) and when they cannot claim any other provision or benefit. Additionally, they should not be residing in prison or in a detention house. Individuals may claim disability benefits when they do not have any working capacity, and are not able to develop any working capacity in the long term.

Employment conditions

Employment contract. Employment contract was measured by asking participants under which type of contract they were working (permanent contract, temporary contract, and temporary employment agency contract). A permanent contract is a contract for an undetermined period of time. A permanent contract is a contract for an undetermined period of time. A temporary contract is a contract that ends after a fixed period of time by operation of law. A temporary employment agency contract is a contract with an employment agency. The employment agency receives orders from potential clients, for which the employee is deployed. When the client ends the order, the temporary employment agency contract ends. When individuals are in a temporary contract with an employment agency for 78 weeks (interruptions below six months are permitted), they will receive a contract for a fixed period of time (a temporary contract).

Work hours per week. Work hours per week was obtained by asking how many hours on average the individual worked. In the Netherlands, 12-35 work hours refers to part-time work and > 35 hours refers to full-time work[8].

Monthly income. Monthly income (300 euro or less, between 300 and 600 euro, between 301 and 600 euro, between 601 and 900 euro, between 901 and 1200 and 1200 euro or higher) was obtained by asking individuals what their average monthly income is. Categories above 1200 were collapsed due to data sparsity.

Supplement 3 – Results latent class analysis

Table S2. Global fit statistics for labour market outcome groups (latent classes)

Model	Number of Parameters	Log-likelihood	BIC	AIC	Entropy
Two	24	-7296.93	14769.77	14641.87	0.99
Three	33	-6957.32	14156.50	14013.64	0.89
**Four**	**47**	-6866.53	14040.88	13859.06	0.90
Five	51	-6814.74	14003.26	13782.48	0.86
Six	60	-6796.77	14033.29	13713.54	0.87

Note: Direct effects between monthly income and educational status, and between work hours and monthly income, were included in the four class solution to fulfill assumptions of local independence. After inclusion of these direct effects, the model fit of the four class solution further improved (Npar: 44; LL: -6811,8576; BIC: 13946,1953; AIC: 13711,7152; Entropy: 0.93. BIC = Bayesian Information Criterion. AIC = Akaike Information Criterion

Table S3. Labour market outcome variables at age 22, conditional on labour market outcome groups (six-class solution)

Variable	Students with side-jobs, N = 423 (%) [a]	Non-working students, N = 386 (%)[a]	Low income early workers, N = 255 (%)[a]	Students with part-time jobs, N = 203 (%)[a]	High income early workers, N = 172 (%)[a]	Inactive individuals, N = 85 (%)[a]
Educational status						
Not in education	0.07	0.15	0.83	0.03	0.87	0.78
Lower education	0.00	0.01	0.01	0.00	0.01	0.01
Vocational education	0.13	0.16	0.07	0.11	0.06	0.09
Applied science or university	0.79	0.69	0.09	0.86	0.06	0.13
Employment status						
Unemployed	0.00	0.99	0.00	0.00	0.00	0.99
Employed with permanent contract	0.50	0.00	0.46	0.45	0.45	0.00
Employed with temporary contract	0.31	0.00	0.37	0.39	0.28	0.00
Employed with temporary employment agency contract	0.16	0.01	0.15	0.12	0.20	0.00
Self-employed	0.04	0.00	0.02	0.04	0.07	0.01
Benefit status	0.00	0.00	0.07	0.00	0.02	0.94
Work hours/ week						
0 hours	0.00	1.00	0.00	0.00	0.00	1.00
Between 1 and 12 hours (side-job)	0.97	0.00	0.04	0.02	0.00	0.00
Between 12 and 35 hours (part-time)	0.02	0.00	0.88	0.83	0.10	0.00
>35 hours (full-time)	0.00	0.00	0.08	0.15	0.90	0.00
Monthly income in Euro						
<300	0.25	0.30	0.03	0.10	0.00	0.05
300-601	0.39	0.39	0.15	0.29	0.01	0.19
601-900	0.26	0.22	0.30	0.33	0.06	0.31
901-1200	0.09	0.07	0.33	0.21	0.28	0.30
> 1200	0.02	0.01	0.19	0.06	0.66	0.15

[a] conditional on group membership

Table S4. Parameter estimates (gamma coefficients and their standard errors) for associations between adverse experiences between ages 0 and 16 across the four labour market outcome groups at age 22 (six-class solution)

Variable	Students with side-jobs, N = 423 (%) [a]	Non-working students, N = 386 (%)[a]	Low income early workers, N = 255 (%)[a]	Students with part-time jobs, N = 203 (%)[a]	High income early workers, N = 172 (%)[a]	Inactive individuals, N = 85 (%)[a]
**Peer influences**						
Bullying victimisation	-0.31 (0.11)	-0.09 (0.11)	-0.08 (0.14)	-0.09 (0.14)	-0.05 (0.15)	0.63 (0.13)
Peer rejection	-0.28 (0.13)	0.01 (0.12)	0.08 (0.15)	-0.15 (0.18)	-0.27 (0.20)	0.60 (0.14)
**Loss or threat of loss**						
Familial death	0.01 (0.15)	-0.26 (0.20)	-0.17 (0.25)	0.07 (0.21)	-0.12 (0.27)	0.47 (0.20)
Illness of a sibling	-0.03 (0.09)	0.21 (0.09)	-0.09 (0.13)	-0.03 (0.13)	-0.04 (0.14)	-0.02 (0.15)
Illness of a parent	-0.03 (0.06)	-0.07 (0.06)	0.07 (0.08)	0.02 (0.09)	-0.09 (0.09)	0.11 (0.10)
**Material deprivation**						
Parental unemployment	-0.03 (0.09)	0.03 (0.09)	-0.04 (0.12)	-0.13 (0.13)	-0.12 (0.13)	0.28 (0.13)
Financial difficulties	0.06 (0.13)	0.13 (0.13)	0.32 (0.16)	-0.46 (0.31)	-0.48 (0.24)	0.43 (0.15)
**Family dynamics**						
Familial conflicts	-0.15 (0.11)	-0.05 (0.11)	0.24 (0.13)	-0.39 (0.22)	-0.31 (0.19)	0.66 (0.14)
Parental divorce	-0.25 (0.08)	-0.06 (0.07)	0.04 (0.09)	-0.20 (0.11)	-0.01 (0.09)	0.48 (0.10)
Parental mental health	-0.01 (0.07)	0.00 (0.07)	-0.09 (0.09)	-0.04 (0.10)	-0.12 (0.10)	0.25 (0.11)
Parental addiction	-0.01 (0.15)	-0.05 (0.16)	-0.11 (0.22)	-0.65 (0.31)	0.14 (0.19)	0.68 (0.17)
**Maltreatment**						
Sexual abuse	-0.17 (0.13)	0.07 (0.12)	-0.02 (0.16)	-0.47 (0.22)	0.05 (0.16)	0.54 (0.14)
Physical abuse	-0.26 (0.13)	-0.03 (0.12)	-0.10 (0.16)	-0.01 (0.17)	-0.24 (0.17)	0.63 (0.14)
Emotional abuse	-0.25 (0.08)	-0.05 (0.08)	-0.02 (0.11)	-0.02 (0.11)	0.01 (0.10)	0.33 (0.11)

Notes. Row estimates sum up to approximately 0 (slight deviations are due to rounding). Presented coefficients are for exposure (1 = exposed). To obtain coefficients for the reverse (0 = non-exposed) the respective sign for each estimate is to be reversed. Estimates within categories (1 = exposed and 0 = non-exposed) sum up to zero. Positive coefficients indicate that a combination of variables (here: exposure to a respective AE and group membership) is more likely to occur than average; negative coefficients refer to the opposite.

Supplement 4 – Missing data overview

Table S5. Number and percentage of missing values for adverse experiences, labour market participation states and employment conditions

Variables (N, %)	
Bullying	43 (2.8%)
Peer rejection	57 (3.74%)
Familial death	0 (0.0%)
Parental illness	32 (2.1%)
Parental mental health problems	82 (5.4%)
Parental addiction	58 (3.8%)
Sibling illness	41 (2.7%)
Parental unemployment	332 (21.8%)
Parental divorce	29 (1.9%)
Financial difficulties	110 (7.2%)
Familial conflicts	111 (7.3%)
Sexual abuse	98 (6.4%)
Emotional abuse	96 (6.3%)
Physical abuse	101 (6.6%)
Educational status	0 (0.0%)
Employment status	6 (0.4%)
Hours work/ week	4 (0.3%)
Receiving benefits	27 (1.8%)
Monthly income in Euro	61 (4.0%)

1 Achenbach TM, Rescorla LA. *Manual for the ASEBA school-age forms & profiles*. Burlington, VT: : University of Vermont, Research Center for Children, Youth & Families 2001.

2 Ormel J, Oldehinkel AJ, Ferdinand RF, *et al.* Internalizing and externalizing problems in adolescence: general and dimension-specific effects of familial loadings and preadolescent temperament traits. *Psychol Med* 2005;**35**:1825–35. doi:10.1017/S0033291705005829

3 Sijtsema JJ, Oldehinkel AJ, Veenstra R, *et al.* Effects of structural and dynamic family characteristics on the development of depressive and aggressive problems during adolescence. The TRAILS study. *Eur Child Adolesc Psychiatry* 2014;**23**:499–513. doi:10.1007/s00787-013-0474-y

4 Bouma EMC, Ormel J, Verhulst FC, *et al.* Stressful life events and depressive problems in early adolescent boys and girls: The influence of parental depression, temperament and family environment. *J Affect Disord* 2008;**105**:185–93. doi:10.1016/j.jad.2007.05.007

5 Oldehinkel AJ, Ormel J, Veenstra R, *et al.* Parental Divorce and Offspring Depressive Symptoms: Dutch Developmental Trends During Early Adolescence. *J Marriage Fam* 2008;**70**:284–93. doi:10.1111/j.1741-3737.2008.00481.x

6 Amone-P'olak K, Ormel J, Huisman M, *et al.* Life Stressors as Mediators of the Relation Between Socioeconomic Position and Mental Health Problems in Early Adolescence: The TRAILS Study. *J Am Acad Child Adolesc Psychiatry* 2009;**48**:1031–8. doi:10.1097/CHI.0b013e3181b39595

7 Oldehinkel AJ, Ormel J. A longitudinal perspective on childhood adversities and onset risk of various psychiatric disorders. *Eur Child Adolesc Psychiatry* 2015;**24**:641–50. doi:10.1007/s00787-014-0540-0

8 Central Bureau of Statistics. Arbeidsdeelname; kerncijfers. 2022.https://www.cbs.nl/nl-nl/cijfers/detail/82309NED?q=deeltijd definitie (accessed 14 Mar 2022).
